# Comparison of the Effects of Sevoflurane and Isoflurane on Postoperative Cognitive Dysfunction Following Laparoscopic Cholecystectomy: A Prospective Study

**DOI:** 10.7759/cureus.89140

**Published:** 2025-07-31

**Authors:** Supratik Ganguly, Azizul Haque, Barun Ram, Mukesh Kumar, Ladhu Lakra

**Affiliations:** 1 Department of Anaesthesiology, Rajendra Institute of Medical Sciences, Ranchi, IND; 2 Department of Trauma and Critical Care, Rajendra Institute of Medical Sciences, Ranchi, IND; 3 Department of Cardiac Anaesthesia, Rajendra Institute of Medical Sciences, Ranchi, IND

**Keywords:** cholecystectomy, isoflurane, laparoscopy, moca scores, sevoflurane

## Abstract

Background

Laparoscopic surgeries, including cholecystectomy, are typically conducted with intravenous induction using anaesthetics such as propofol. This study used sevoflurane and isoflurane as alternatives to propofol to minimise adverse events and compare their effectiveness.

Materials and methods

The research was a prospective randomised study conducted at the Department of Anaesthesiology, Rajendra Institute of Medical Sciences (RIMS), Ranchi, Jharkhand, India, for one and a half years. A total of 90 patients participated in the study, evenly split into two groups (sevoflurane and isoflurane) in a 1:1 ratio. Ethical clearance was obtained from the Institutional Ethics Committee (IEC), RIMS, Ranchi, Jharkhand, India, under letter number 131/IEC/RIMS, dated 27 June 2023.

Results

In terms of the American Society of Anesthesiologists (ASA) classification, more participants were categorised as grade II in the sevoflurane and isoflurane groups. Montreal Cognitive Assessment (MoCA) scores were evaluated at various time intervals. There was no significant difference between the sevoflurane and isoflurane groups after six hours of anaesthesia. Total MOCA scores were found to be significant at a p-value of 0.04 after 24 hours of anaesthesia between the sevoflurane and isoflurane groups.

Conclusion

The study concluded that the incidence of the MoCA score decreased in the isoflurane and sevoflurane groups at one hour and six hours postoperatively. Adverse events, such as nausea, vomiting, and shivering, also decreased over time.

## Introduction

Laparoscopic surgery was introduced in the mid-1950s, which at that period was revolutionary due to advantages such as lower overall medical expenses, less blood, fewer pulmonary and surgical problems after surgery, and quicker recovery. Laparoscopic procedures, including laparoscopic cholecystectomy, have been carried out under general anaesthesia (GA) in the past, since GA is the most effective way to treat the breathing abnormalities brought on by the pneumoperitoneum, which includes upward displacement of the diaphragm and reduction in functional residual capacity (FRC), potentially impacting respiratory function [1]. Anaesthetic induction in laparoscopic cholecystectomy is commonly achieved by an intravenous induction agent such as propofol. Inhaled anaesthetics are widely employed for maintaining GA, offering a state of unconsciousness and amnesia when a small quantity of volatile anaesthetic is added to the inspired gas mixture. When used in conjunction with intravenous adjuvants like opioids, benzodiazepines, and muscle relaxants, a balanced anaesthetic approach is attained, providing analgesia, amnesia, and muscle relaxation [2].

The popularity of inhaled anaesthetics stems from their straightforward administration and the capability to monitor their effects reliably through clinical signs and end-tidal concentration. Inhalational agents commonly used are sevoflurane or isoflurane. Sevoflurane is a halogenated inhalational agent containing fluorine. For both adult and paediatric patients, sevoflurane is a great option for a smooth and quick inhalational induction due to its low pungency and quick rise in alveolar anaesthetic concentration [3].

Unlike isoflurane, sevoflurane acts quickly and has a poor blood gas solubility, which causes the alveolar concentration to drop quickly and causes a rapid emergence [3]. Inhaled volatile anaesthetics continue to be the preferred agents for maintaining GA, primarily due to their predictable intraoperative and recovery properties. Effective management of intraoperative haemodynamic stability and early recovery is considered for a balanced anaesthetic technique. Sevoflurane lowers arterial blood pressure, systemic vascular resistance, respiration, and myocardial contractility in a modest way. Isoflurane decreases systemic vascular resistance and lowers arterial blood pressure due to mild beta-adrenergic stimulation. Isoflurane also causes a more pronounced fall in minute ventilation than sevoflurane, causing respiratory depression. Isoflurane, on the other hand, has a slower onset of action and emergence is slower compared to sevoflurane [3].

The goal of a quick induction and recovery is linked to advantages like shorter recovery room stays, quicker operating room turnover times, and earlier home discharges. Notably, the past 15 years have witnessed a significant surge in the trend towards delivering cost-effective care in medical practice. Postoperative cognitive dysfunction (POCD) is defined as a new cognitive impairment arising after a surgical or anaesthetic procedure. Its diagnosis requires both pre- and postoperative psychometric testing [4]. The occurrence of POCD has been well-documented, with rates in the first week after GA and noncardiac surgery in older patients ranging from 18% to 32% [5-9].

The purpose of the Montreal Cognitive Assessment (MOCA) was to rapidly test for minor cognitive impairment [10]. It evaluates a variety of cognitive domains, including conceptual thinking, computations, executive processes, language, attention and focus, memory, and visual-constructional skills. There is a 30-point maximum possible score. A normal score is one of 26 or higher [10].

This study aimed to compare the effects of sevoflurane and isoflurane on POCD following laparoscopic cholecystectomy. Also, characteristics such as tear, sweat, heart rate, and blood pressure were evaluated in both the respective groups.

## Materials and methods

Study design

The study was a prospective, randomised study conducted at the Department of Anaesthesiology, Rajendra Institute of Medical Sciences (RIMS), Ranchi, Jharkhand, India. It lasted one and a half years, from June 2023 to January 2024.

Study population

The study enrolled 90 participants, split evenly into two groups of 45. To qualify, patients had to provide written informed consent, possess an American Society of Anesthesiologists (ASA) physical status I or II, and fall within the age range of 18-60 years across all genders. Exclusion criteria included individuals who declined participation, were younger than 18 or older than 60 years, possessed an ASA physical status III or higher, did not speak Hindi or English, lacked an elementary school education or had a history of significant brain surgery, recent stroke, cognitive impairment, mental dysfunction, schizophrenia, dementia or severe anxiety. A normocarbia was also maintained, ensuring an end-tidal CO_2_ of 35-40 mm Hg.

Data collection

The data that were collected included patients' demographics such as age, gender, height, weight, and MOCA scoring parameters at different intervals, such as preoperatively, one hour after anaesthesia, six hours after anaesthesia, and 24 hours after anaesthesia. Adverse events were also noted after one hour, six hours, and 24 hours. Several other parameters, such as systolic blood pressure (SBP), heart rate (HR), tear, and sweat, were also illustrated at different time intervals between the two groups. Lacrimation (tear production) was measured using Schirmer's test. Perspiration was measured by analysing sweat samples with the use of wearable sensors.

Study procedure

Patients received a maintenance dose of rocuronium at 0.15 mg/kg of body weight to ensure sufficient muscle relaxation. Near the end of the surgery, local anaesthetic infiltration with 0.5% w/v bupivacaine was administered before closing the port site. Inhalational agents, such as sevoflurane or isoflurane, were discontinued after the skin was closed. A reversal agent, neostigmine at 50-70 mcg/kg of body weight, along with an injection of glycopyrrolate 0.0125 mg/kg IV, was given, and patients were extubated with appropriate oral suctioning. They were then transferred to the post-anaesthesia care unit for continued monitoring.

An assistant, blinded to the intraoperative inhalational gas assignments, collected all postoperative data. The cessation of the inhalation agent marks the conclusion of GA. MoCA assessments were performed one, six, and 24 hours following GA. Patients would be excluded from the study if the surgery was not completed in time to obtain the six-hour MoCA before 10 pm to prevent the effects of circadian rhythms on alertness. Sleepiness can impair cognitive function, potentially lowering the MoCA score. A decrease greater than two points from the MoCA baseline was considered significant. The occurrence of postoperative nausea and vomiting was recorded following endotracheal extubation and again before each MoCA assessment. Additionally, the intensity of postoperative shivering was documented following extubation.

Statistical analysis

Data were summarised as means and standard deviations for numerical variables and counts and percentages for categorical variables. For statistical analysis, the data were entered into a Microsoft Excel spreadsheet (Microsoft Corporation, Redmond, WA) and analysed using SPSS software (IBM Corp., Armonk, NY). The chi-squared test was applied to qualitative data to compare proportions for paired values. The Student’s t-test was employed for quantitative data, with results represented as the mean and standard deviation of the differences between paired means. A p-value <0.05 was deemed significant.

Ethical clearance

Ethical clearance was obtained from the Institutional Ethics Committee (IEC), RIMS, Ranchi, Jharkhand, India, under letter number 131/IEC/RIMS, dated 27 June 2023. The study was registered on the Clinical Trials Registry - India (CTRI) under the registration number (CTRI/2024/03/063503), dated 4 March 2024.

## Results

Table 1 represents patient demographics such as age, gender (male and female), height, weight, and ASA categorisation among participants. Most of the patients in the sevoflurane group were male, while most of the patients in the isoflurane group were female, with no statistical difference in gender between the two groups. No significant difference was observed in any characteristics between the sevoflurane and isoflurane groups, respectively. As per the ASA categorisation, study subjects were divided into two groups of respective grades I and II. No significant association in the ASA grading has been found between the groups of sevoflurane and isoflurane, respectively.

**Table 1:** Patient demographics. Data are presented as either mean ± SD or n (%). P-value was considered significant at <0.05. An independent t-test or chi-square test was used to obtain the p-value. ASA: American Society of Anesthesiologists.

Characteristics	Sevoflurane (n = 45)	Isoflurane (n = 45)	t-statistic value/chi-square value	p-value
Age (in years)	35.7±9.14	37.3±9.32	0.82	0.413
Male	27 (60%)	23 (51.1%)	-0.8	0.19
Female	18 (40%)	22 (48.9%)	0.84	0.19
Weight (in kgs)	73.7±8.49	74±7.45	0.17	0.85
Height (in cms)	168.8±5.54	166.6±7.04	-1.64	0.10
ASA grading				
Grade I	19 (42.2%)	14 (31.1%)	1.19	0.274
Grade II	26 (57.8%)	31 (68.9%)		

The MOCA scores were assessed at different time intervals. Table 2 represents MOCA scoring at different intervals, such as preoperatively, one hour after anaesthesia, six hours after anaesthesia, and 24 hours after anaesthesia, respectively. Preoperatively, total MOCA scores were 26.71 ± 0.96 in the sevoflurane group and 26.62 ± 0.65 in the isoflurane group, respectively. MOCA scores after one hour of anaesthesia were 8.80 ± 1.70 in the sevoflurane group and 9.93 ± 1.51 in the isoflurane group, respectively. Statistically significant difference was observed between both the sevoflurane and isoflurane groups after one and 24 hours of anaesthesia with a p-value less than 0.05. There was no significant difference between the sevoflurane and isoflurane groups after six hours of anaesthesia. Total MOCA scores were found to be significant at a p-value of 0.04 after 24 hours of anaesthesia between the sevoflurane and isoflurane groups.

**Table 2:** Montreal Cognitive Assessment (MoCA) scoring at different time intervals. Data are presented as either mean ± SD or n (%). P-value was considered significant at <0.05. An unpaired t-test was used to obtain the p-value.

Parameters	Pre-operative				1 hour after anaesthesia				6 hours after anaesthesia				24 hours after anaesthesia			
	Sevoflurane (n = 45)	Isoflurane (n = 45)	t-statistic value	p-value	Sevoflurane (n = 45)	Isoflurane (n = 45)	t-statistic value	p-value	Sevoflurane (n = 45)	Isoflurane (n = 45)	t-statistic value	p-value	Sevoflurane (n = 45)	Isoflurane (n = 45)	t-statistic value	p-value
Executive (5)	4.56±0.54	4.51±0.54	-0.43	0.66	0.01±0.1	0.02±0.14	0.39	0.69	4.47±0.50	4.47±0.50	0.000	1.000	4.58±0.49	4.64±0.48	0.58	0.55
Naming (3)	2.71±0.45	2.62±0.49	-0.12	0.90	1.07±0.83	1.51±0.66	2.78	0.006	2.4±0.53	2.58±0.49	1.67	0.09	2.58±0.49	2.71±0.45	1.31	0.19
Attention (6)	4.58±0.62	4.38±0.49	-1.6	0.09	1.27±0.68	1.69±0.46	3.43	<0.001	4.11±0.61	4.04±0.47	-0.61	0.54	4.22±0.59	4.36±0.48	1.23	0.22
Language (3)	2.02±0.39	2.09±0.51	0.73	0.46	0.02±0.14	0.01±0.1	0.39	0.69	1.82±0.49	1.96±0.20	1.77	0.07	1.98±0.26	1.98±0.14	0.00	1.000
Abstraction (2)	1.91±0.28	1.89±0.31	-0.32	0.74	0.02±0.14	0.01±0.1	0.39	0.69	1.6±0.65	1.8±0.4	1.75	0.08	1.91±0.28	1.78±0.42	-1.72	0.08
Delayed recall (5)	4.33±0.60	4.60±0.53	2.26	0.02	1.44±0.65	1.16±0.79	-1.83	0.06	4.27±0.68	4.38±0.57	0.83	0.40	4.47±0.58	4.71±0.45	2.19	0.03
Orientation (6)	5.82±0.38	5.89±0.31	0.95	0.34	4.22±1.12	4.89±1.11	2.85	0.005	5.49±0.5	5.58±0.58	0.78	0.43	5.73±0.44	5.78±0.42	0.55	0.58
Education < 12 years (1)	0.78±0.42	0.64±0.48	-1.4	0.14	0.76±0.43	0.67±0.47	-0.94	0.34	0.76±0.43	0.67±0.47	-0.94	0.34	0.76±0.43	0.64±0.48	-1.24	0.21
Total MoCA (30)	26.71±0.96	26.62±0.65	-0.52	0.60	8.80±1.70	9.93±1.51	3.33	0.001	24.91±1.6	25.47±1.21	1.87	0.06	26.22±1.12	26.6±0.75	1.99	0.04

Table 3 presents the adverse postoperative events, including nausea, vomiting, and shivering, recorded at one, six, and 24 hours throughout the study.

**Table 3:** Postoperative adverse events. Data are presented as n (%). P-value was considered significant at <0.05. An unpaired t-test was used to obtain the p-value.

Adverse events	After 1 hour				After 6 hours				After 24 hours			
	Sevoflurane (n = 45)	Isoflurane (n = 45)	t-value	p-value	Sevoflurane (n = 45)	Isoflurane (n = 45)	t-value	p-value	Sevoflurane (n = 45)	Isoflurane (n = 45)	t-value	p-value
Nausea and vomiting	08 (17.8%)	06 (13.3%)	-0.58	0.28	07 (15.6%)	00	-	-	01 (2.2%)	01 (2.2%)	0	0.5
Shivering	05 (11.1%)	04 (8.9%)	-0.35	0.36	02 (4.4%)	02 (4.4%)	0	0.5	01 (2.2%)	0	-	-

Table 4 shows pressure, rate, sweating, and tears (PRST) scores at different time intervals among participants. Scores after induction were higher in the sevoflurane group compared to the isoflurane group. There was no significant difference observed between groups at different time intervals.

**Table 4:** PRST scores at different time intervals. Data are presented as mean ± SD. P-value was considered significant at <0.05. An unpaired t-test was used to obtain the p-value. PRST: pressure, rate, sweating, and tears.

Time intervals	Groups	PRST scores	P-value
Before induction	Sevoflurane (n = 45)	0.0±0.0	-
	Isoflurane (n = 45)	0.0±0.0	
After induction	Sevoflurane (n = 45)	1.87±1.01	0.60
	Isoflurane (n = 45)	1.76±1.0	
After 15 minutes	Sevoflurane (n = 45)	1.82±1.09	0.34
	Isoflurane (n = 45)	2.04±1.10	
After 30 minutes	Sevoflurane (n = 45)	1.84±1.22	0.57
	Isoflurane (n = 45)	1.71±0.99	

## Discussion

This study assessed early POCD following laparoscopic cholecystectomy and its prompt management. It also compared the incidence of postoperative shivering and nausea and vomiting between the two patient groups. Results indicated that both groups, each consisting of 45 patients, had comparable baseline characteristics, including mean age, gender distribution, height, weight, ASA I and II classification, and preoperative MoCA scores. The overall mean MoCA score at baseline was above 26, suggesting that participants in both groups did not exhibit cognitive dysfunction. However, at the six-hour postoperative mark, total MoCA scores dropped in both the sevoflurane and isoflurane groups. The scores at this point were below 26, indicating cognitive dysfunction. The total MoCA scores were found to be statistically significant with a p-value of <0.001 between both the groups of sevoflurane and isoflurane one hour post operation. No significant differences were noted preoperatively, at six hours, or 24 hours.

In 2019, Peng et al. conducted a single-centre prospective observational study to evaluate POCD in elderly patients undergoing major cardiac surgeries, using the Mini-Mental State Examination (MMSE). The study found a statistically significantly higher incidence of POCD in the isoflurane group at all three postoperative time points: day one, three, and five [11]. Additionally, this study employed a different POCD assessment tool and included elderly participants more prone to cognitive dysfunction [12,13].

A study conducted by Hadzimesic et al. in 2011-2012 compared the effects of propofol, isoflurane, and sevoflurane on the rate of cognitive function recovery in elderly patients undergoing lumbar microdiscectomy due to herniated lumbar discs. The researchers included only patients in the ASA I category in their study. They employed the orientation-memory-concentration test to assess postoperative cognitive recovery at one, five, and 10 minutes post surgery. After one minute of extubation, the researchers found a statistically significant difference between the sevoflurane and isoflurane groups, with a p-value of 0.049. Isoflurane also demonstrated a slower recovery of cognitive function compared with sevoflurane [14].

In their study, Gang et al. explored the effects of isoflurane versus sevoflurane on postoperative cognitive function while measuring serum β-amyloid levels in elderly patients undergoing abdominal surgery. They assessed cognitive dysfunction using the MMSE one day before surgery and again three days and 12 months postoperatively, alongside evaluating blood parameters. In contrast to our findings, Gang et al. reported that the isoflurane group exhibited significantly more cognitive dysfunction than the sevoflurane group (40.6% vs. 33.3%) on the 3rd day after surgery. However, after 12 months, this discrepancy was no longer evident, suggesting no long-term differences in cognitive dysfunction between the two groups [15].

Pensado Castiñeiras et al. [16] conducted a clinical study involving 75 participants to compare anaesthetic recovery and psychomotor function in patients undergoing prolonged urological surgery. These patients were randomised into three groups: desflurane, sevoflurane, and isoflurane. The Newman-Trieger and Aldrete tests were applied during the first 24 hours of surgery. However, the authors found no significant differences in the psychomotor recovery among the three groups. This finding is concordant with that of our study. Furthermore, the present study did not identify any statistically significant difference in postoperative nausea and vomiting between the isoflurane and sevoflurane groups, a finding consistent with Castineiras’s study.

Parsahomavandi et al. [17] conducted a clinical study involving isoflurane and sevoflurane to compare the incidence of postoperative shivering and its severity. The participants were allocated to two groups, each comprising 40 patients. The sevoflurane group exhibited less shivering at five and 15 minutes postoperatively compared with the isoflurane group. The severity of postoperative shivering was also higher in the isoflurane group than in the sevoflurane group. Our study reported no difference in the incidence of postoperative shivering between the isoflurane and sevoflurane groups. The present study showed no significant differences in PRST scores at different time intervals in both the groups of sevoflurane and isoflurane, respectively.

This study has a number of limitations. First, the study was performed only at a single centre with a small sample size. To validate the findings, further multicentric studies involving diverse age groups and clinical profiles are needed. Second, due to limited resources, more recent tools for detecting cognitive dysfunction could not be used. Future research should also assess the incidence of POCD immediately after surgery and in the later postoperative period.

## Conclusions

This prospective, randomised study concluded that the incidence of the MoCA score decreased in the isoflurane and sevoflurane groups at one hour and six hours postoperatively. While the incidence of POCD was not statistically different between the two groups, isoflurane demonstrated superior results in attention, orientation, and naming components of the MoCA scoring after the first hour, along with a marginally improved total MoCA score. Additionally, there was a notable decrease in adverse events over time.
